# The effect of corneal cross-linking on the anterior and posterior parameters of the cornea: A prospective repeatability study

**Published:** 2019

**Authors:** Hassan Hashemi, Shiva Mehravaran, Soheila Asgari

**Affiliations:** * Noor Research Center for Ophthalmic Epidemiology, Noor Eye Hospital, Tehran, Iran; ** ASCEND Center for Biomedical Research, Morgan State University, Baltimore, MD, USA; *** Noor Ophthalmology Research Center, Noor Eye Hospital, Tehran, Iran

**Keywords:** corneal cross-linking, corneal index, reliability study

## Abstract

**Objective.** To determine the effect of corneal cross-linking (CXL) on the anterior and posterior corneal indices in terms of their repeatability and change as measured with Pentacam.

**Methods.** Thirty eyes of 30 patients with progressive keratoconus undergoing CXL were enrolled. At each visit (pre-CXL, 6 and 12 months after CXL), imaging were done twice, one hour apart to determine the repeatability index (RI) and intra-class correlation coefficient (ICC). For same session measurements, we computed the intra-session repeatability. We also calculated 4 measures of change by subtracting baseline from 1-year results and determined the repeatability of measures of change.

**Results.** There was no significant difference between the intra-session RI at baseline, 6 months, and 12 months for anterior Kmax-3mm (P=0.609), anterior Kmin-3mm (P=0.548), Kmax-8mm (P=0.860), posterior Kmax-3mm (P=0.717), posterior Kmin-3mm (P=0.548), Q-value-6mm (P=0.890), central corneal thickness (P=0.751), minimum corneal thickness (P=0.787), or anterior chamber depth (P=0.760). The ICCs for these indices were higher than 0.9. For keratoconus indices, there was no significant difference between the intra-session RI at baseline and follow-ups (P>0.05), and the ICC were higher than 0.9 except for baseline and 6-month index of height asymmetry (IHA). The ICC for all 1-year measures of change were more than 0.75 except for posterior corneal indices and IHA.

**Conclusion.** Pentacam repeatability of different indices is not affected by CXL. However, the change of indices showed high variance, which should be taken into consideration, especially in systematic reviews because inter-study differences can be due to low repeatability of the measures of change.

## Introduction

Corneal cross-linking (CXL) is an effective therapeutic approach to halt corneal steepening and thinning in keratoconus patients [**1**,**2**]. The effectiveness of different protocols of this procedure has been shown in multiple studies examining results of clinical examinations and changes in important corneal indices such as pachymetry and keratometry [**3**,**4**]. Among accelerated CXL methods, the protocol applying 18mW/ cm2 for 5 minutes has shown variable effectiveness results in different studies. Some studies have reported a decrease in maximum keratometry (Kmax) after CXL [**5**,**6**], while some suggest they tend to remain stable [**3**,**7**]. Similarly, some studies suggest that corneal thickness (CT) decreases after CXL [**3**,**5**], while CT increase [**6**] and stability [**7**] have been reported as well.

Among diagnostic modalities, Pentacam (Oculus Optikgerate GmbH, Germany), which utilizes Scheimpflug imaging technology, is capable of analyzing the anterior and posterior cornea separately and is commonly used in clinical settings. Several studies have been conducted to examine the repeatability of anterior [**8**] and posterior [**9**] keratometry, corneal thickness [**10**] and aberrations [**9**] in keratoconus patients; some studies have found the repeatability acceptable [**10**], and some suggest it is acceptable only for mild keratoconus (with Kmax < 55.0D) [**8**]. Although the repeatability of measurements with this device after CXL has been examined and reported [**9**], the repeatability of measurements has not been compared between keratoconus patients and the post-CXL groups. To determine the potential effect of CXL on Pentacam repeatability, it is necessary to evaluate a patient group before and after CXL. The present study was conducted with this objective. Results should help us get a better understanding of CXL-related effects on the repeatability and precision of the device, and they can serve as a guide for clinicians to evaluate treatment effectiveness.

## Methods

This prospective study was conducted in 2015 at the Keratoconus Clinic of Noor Eye Hospital in Tehran and approved by the Institutional Review Board of Noor Ophthalmology Research Center. The study adhered to the Helsinki Declaration at all stages, and all participants signed a written informed consent. Thirty eyes of 30 patients with progressive keratoconus (at least one diopter (D) increase in maximum keratometry (Kmax), manifest cylinder, or manifest refraction spherical equivalent, and loss of at least 2 lines of corrected distance visual acuity over the past 12 months), aged 15-35 years, Kmax < 55.0D, and minimum corneal thickness (MCT) > 400 μm with no history of eye surgery were enrolled in the study. We selected patients with keratoconus grade I to III based on Pentacam indexes, the index of surface variance (30≤ISV≤90), and the keratoconus index (1.07≤KI≤1.25) [**11**].

**The CXL procedure**

After administering local anesthesia using proparacaine hydrochloride 0.5% eye drops, the central 9mm of the corneal epithelium was manually removed. After removing the lid speculum, riboflavin 0.1% drop in 20% dextran (Streuli Pharmaceuticals, Uznach, Switzerland) was instilled on the corneal surface every 3 minutes for half an hour. After anterior chamber saturation with riboflavin, irradiation was performed using CCL 365 (PESCHKE Meditrade GmbH, Waldshut-Tiengen, Germany) at an intensity of 18mW/ cm2. During irradiation, riboflavin instillation was repeated every 3 minutes. At the end of this step, the corneal surface was rinsed with sterile balanced saline solution, a soft bandage contact lens (Night & Day, Ciba Vision, Duluth, GA, USA) was placed on the cornea, and levofloxacin eye drops were instilled. The post-CXL regimen included levofloxacin eye drop four times daily, betamethasone 0.1%, and Hypromellose preservative free artificial tears as required. Patients were examined on days 1 and 3 after CXL. The lens was removed after observing epithelial healing. After removing the lens, levofloxacin was discontinued and betamethasone was continued for 1 week, 4 times a day. If the epithelial healing was not complete, daily visits continued until complete re-epithelialization was observed. There was no complication during or after CXL.

**Measurement protocol**

All examinations were performed between 9 am and noon. In addition to routine ophthalmic examinations and measurements of visual acuity and refraction, all patients were examined with Pentacam HR (Oculus, Inc., Lynnwood, WA; software version6.03r19, data management version1.18r08). Imaging was repeated, if necessary, until the image status was stated as OK. Before the test, patients were asked to blink several times. The second acquisition at each session was done at a one-hour interval. The same technician performed all imaging at baseline, and at 6 and 12 months after CXL.

Of the indexes measured with Pentacam, the 3 mm Kmax and minimum keratometry (Kmin) of the anterior and posterior cornea, 8 mm Kmax, central corneal thickness (CCT), MCT, anterior chamber depth (ACD), and keratoconus indices including index of surface variation (ISV), index of vertical asymmetry (IVA), keratoconus index (KI), central keratoconus index (CKI), index of height asymmetry (IHA), and index of height decentration (IHD) were extracted and analyzed.

**Statistical analysis**

To evaluate the repeatability of the measurements, the intra-class correlation coefficient (ICC), and the repeatability index (RI) were calculated for paired measurements of each of the three visits (intra-session repeatability). To calculate RI, the standard deviation of the subject (Sw) was multiplied by 2.77. Lower values of this index indicated better repeatability due to less test-retest variation [**12**]. To evaluate the effect of CXL on the repeatability of the measurements, the RIs were compared using repeated measures analysis of variance (ANOVA).

Using the baseline and 1-year results, we also calculated 4 measures of change for each index and compared them using repeated measures ANOVA. We also determined their ICC and RI to test their repeatability, as well as the standard deviations of the RIs to illustrate their variations.

## Results

The mean age of the participants was 23.5 ± 3.9 (range, 15 to 30) years and 60% were male. 

**Intra-session measurements**

**Table 1** summarizes the first and second measurement at each session before and after CXL. There was no significant difference between baseline, 6-month post-CXL, and 12-month post-CXL values of RI for anterior Kmax-3mm (P=0.609), anterior Kmin-3mm (P=0.548), Kmax-8mm (P=0.860), posterior Kmax-3mm (P=0.717), posterior Kmin-3mm (P=0.548), Q-value (P=0.890), CCT (P=0.751), MCT (P=0.787), or ACD (P=0.760). The ICC was also more than 0.9 for all these indices. 

**Table 1:** Repeatability of corneal parameters measured by Pentacam after accelerated cross-linking

		Take 1	Take 2	Difference ± SD	ICC (CI 95%)	RI	P-value*
Anterior Kmax-3mm (D)	Baseline	48.53±3.90	48.53±3.95	0.00±0.32	0.997 (0.992 to 0.999)	0.48	0.609
	6 M	48.64±4.10	48.68±4.03	0.04±0.34	0.997 (0.991 to 0.999)	0.53	
	12 M	48.47±3.70	48.33±3.57	0.14±0.30	0.997 (0.992 to 0.999)	0.41	
Anterior Kmin-3mm (D)	Baseline	44.90±2.99	44.89±3.12	0.00±0.37	0.993 (0.982 to 0.997)	0.51	0.548
	6 M	44.89±3.26	44.93±3.15	0.04±0.37	0.993 (0.983 to 0.997)	0.48	
	12 M	44.81±3.14	44.84±3.15	0.03±0.30	0.995 (0.989 to 0.998)	0.39	
Anterior Kmax-8mm (D)	Baseline	52.94±6.10	52.98±6.01	0.04±0.61	0.995 (0.988 to 0.998)	0.86	0.860
	6 M	53.21±6.08	53.17±6.04	0.05±0.52	0.996 (0.991 to 0.998)	0.80	
	12 M	52.75±5.86	52.50±5.46	0.25±0.66	0.993 (0.984 to 0.997)	0.91	
Posterior Kmax-3mm (D)	Baseline	7.34±0.80	7.33±0.81	0.01±0.10	0.992 (0.980 to 0.997)	0.16	0.16
	6 M	7.30±0.78	7.34±0.80	0.04±0.10	0.992 (0.979 to 0.997)	0.13	
	12 M	7.27±0.80	7.28±0.8	0.00±0.08	0.995 (0.988 to 0.998)	0.13	
Posterior Kmin-3mm (D)	Baseline	6.54±0.65	6.55±0.66	0.01±0.08	0.993 (0.983 to 0.997)	0.39	0.548
	6 M	6.51±0.65	6.53±0.63	0.02±0.10	0.988 (0.971 to 0.995)	0.48	
	12 M	6.49±0.61	6.49±0.61	0.00±0.10	0.987 (0.969 to 0.995)	0.51	
Q-value	Baseline	-0.79±0.42	-0.77±0.40	0.02±0.07	0.987 (0.968 to 0.995)	0.08	0.890
	6 M	-0.86±0.47	-0.87±0.48	0.01±0.06	0.991 (0.979 to 0.996)	0.09	
	12 M	-0.84±0.46	-0.83±0.48	0.01±0.05	0.994 (0.985 to 0.998)	0.08	
CCT (µm)	Baseline	483.2±36.5	485.5±36.2	2.28±7.75	0.977 (0.945 to 0.991)	11.00	0.751
	6 M	476.9±35.3	476.8±36.2	0.14±6.62	0.983 (0.958 to 0.993)	9.23	
	12 M	476.3±37.0	475.4±35.6	0.90±6.59	0.984 (0.960 to 0.993)	10.35	
MCT (µm)	Baseline	472.7±35.7	475.7±35.9	2.95±7.49	0.978 (0.947 to 0.991)	12.12	0.787
	6 M	467.9±34.3	467.4±36.6	0.48±7.86	0.975 (0.941 to 0.990)	11.19	
	12 M	466.8±36.4	467.6±34.4	0.81±6.95	0.981 (0.953 to 0.992)	10.54	
ACD (mm)	Baseline	3.25±0.26	3.24±0.23	0.00±0.04	0.985 (0.964 to 0.994)	0.05	0.760
	6 M	3.24±0.26	3.22±0.25	0.01±0.06	0.974 (0.938 to 0.990)	0.06	
	12 M	3.23±0.26	3.22±0.26	0.01±0.04	0.989 (0.974 to 0.996)	0.05	
* Comparison of RIs (at baseline, 6 months (M) post-CXL, and 12 months post-CXL) by repeated measures ANOVA							
Kmax = maximum keratometry; D = diopter; Kmin = minimum keratometry; CCT = central corneal thickness; MCT = minimum corneal thickness; ACD = anterior chamber depth							

As presented in **Table 2**, there was no significant difference between baseline, 6-month post-CXL, and 12-month post-CXL values of RI for keratoconus indices (all P > 0.05), and the repeatability of the indices was higher than 0.9, except for pre-CXL and 6-month follow up IHA (P=0.8). 

**Table 2:** Repeatability of keratoconus indices measured with Pentacam after accelerated corneal cross-linking

		Take 1	Take 2	Difference ± SD	ICC (CI 95%)	RI	P-value*
KI	Baseline	1.15±0.08	1.15±0.08	0.00±0.00	0.993 (0.983 to 0.997)	0.01	0.442
	6 M	1.16±0.08	1.15±0.08	0.00±0.01	0.990 (0.976 to 0.996)	0.01	
	12 M	1.15±0.08	1.15±0.08	0.00±0.01	0.993 (0.984 to 0.997)	0.01	
CKI	Baseline	1.05±0.04	1.05±0.04	0.00±0.01	0.981 (0.955 to 0.992)	0.01	0.505
	6 M	1.05±0.04	1.06±0.04	0.00±0.01	0.990 (0.975 to 0.996)	0.01	
	12 M	1.05±0.04	1.05±0.05	0.00±0.00	0.995 (0.989 to 0.998)	0.003	
ISV	Baseline	62.57±25.85	62.43±25.26	0.14±1.68	0.998 (0.995 to 0.999)	1.96	0.518
	6 M	67.14±25.19	66.71±24.95	0.43±1.94	0.997 (0.993 to 0.999)	2.70	
	12 M	65.38±25.55	65.48±25.21	0.09±1.81	0.997 (0.994 to 0.999)	2.61	
IVA	Baseline	0.61±0.31	0.62±0.30	0.01±0.04	0.991 (0.979 to 0.997)	0.05	0.670
	6 M	0.66±0.30	0.65±0.28	0.01±0.04	0.989 (0.973 to 0.996)	0.06	
	12 M	0.64±0.31	0.64±0.31	0.00±0.03	0.995 (0.987 to 0.998)	0.05	
IHA	Baseline	34.56±28.07	35.20±26.64	0.64±13.26	0.883 (0.733 to 0.951)	17.50	0.327
	6 M	29.14±22.23	34.80±22.91	5.67±14.58	0.791 (0.554 to 0.910)	21.32	
	12 M	33.00±22.29	30.90±22.64	2.10±10.03	0.900 (0.771 to 0.958)	12.87	
IHD	Baseline	0.09±0.05	0.09±0.05	0.00±0.01	0.987 (0.968 to 0.995)	0.01	0.745
	6 M	0.09±0.05	0.09±0.05	0.00±0.01	0.986 (0.966 to 0.994)	0.01	
	12 M	0.09±0.05	0.09±0.05	0.00±0.01	0.991 (0.979 to 0.996)	0.01	
* Comparison of RIs (at baseline, 6 months (M) post-CXL, and 12 months post-CXL) by repeated measures ANOVA							
KI = keratoconus index, CKI = center keratoconus index, ISV = index of surface variance, IVA = index of vertical asymmetry, IHA = index of height asymmetry, IHD = index of height decentration							

**Measures of change**

**Table 3** summarizes the measures of change at one year after CXL. Using the two baseline measurements and the two 12-month repeated measurements, 4 measures of change were calculated. Repeated measures ANOVA showed no statistically significant difference between the measures of change for each index (all P > 0.05). Although lower compared to intra-session values, the ICC for all measures of change for keratoconus indices was above 0.75, with the exception of IHA, which was 0.573 (medium repeatability). The ICC values for changes in anterior Kmax-3mm, Kmin-3mm, Kmax-8 mm, and Q-value were 0.864, 0.786, 0.856, and 0.883, respectively. However, for changes in posterior indices, Kmax-3mm and Kmin-3mm, ICC values were even lower (0.546 and 0.643, respectively). The ICC values for changes in CCT, MCT, and ACD were 0.758, 0.804, and 0.748, respectively. As presented in Table 3, the standard deviations of the RIs were almost equal to their respective RI. In other words, there was high variation in the repeatability of the measures of change. 

**Table 3:** Repeatability of 1-year measures of change in corneal parameters measured with Pentacam after accelerated corneal cross-linking

	Baseline take 1		Baseline take 2			
	1-year take 1	1-year take 2	1-year take 1	1-year take 2	ICC (CI 95%)	RI
Anterior Kmax-3mm (D)	-0.06±0.65	-0.06±0.67	-0.20±0.68	-0.20±0.72	0.864 (0.758 to 0.935)	0.56±0.46
Anterior Kmin-3mm (D)	-0.09±0.50	-0.06±0.55	-0.09±0.60	-0.06±0.70	0.786 (0.639 to 0.895)	0.58±0.49
Anterior Kmax-8mm (D)	-0.19±1.39	-0.23±1.18	-0.44±1.52	-0.48±1.32	0.856 (0.746 to 0.931)	1.13±0.93
Posterior Kmax-3mm (D)	-0.08±0.12	-0.06±0.09	-0.08±0.12	-0.06±0.11	0.546 (0.332 to 0.749)	0.19±0.08
Posterior Kmin-3mm (D)	-0.05±0.10	-0.06±0.10	-0.04±0.12	-0.05±0.15	0.643 (0.445 to 0.811)	0.14±0.13
Q-value	+0.04±0.12	+0.03±0.14	+0.07±0.14	+0.06±0.16	0.883 (0.790 to 0.945)	0.10±0.09
CCT (µm)	-6.86±12.26	-7.76±12.04	-9.14±12.09	-10.05±11.34	0.758 (0.598 to 0.879)	13.68±9.19
MCT (µm)	-5.90±14.58	-5.09±13.84	-8.86±12.66	-8.05±12.01	0.804 (0.665 to 0.904)	14.31±8.79
ACD (mm)	-0.02±0.07	-0.02±0.07	-0.01±0.06	-0.02±0.06	0.748 (0.584 to 0.873)	0.07±0.06
KI	-0.01±0.02	-0.01±0.02	-0.00±0.02	-0.00±0.02	0.814 (0.680 to 0.909)	0.02±0.01
CKI	-0.00±0.01	-0.00±0.01	-0.00±0.01	-0.00±0.02	0.893 (0.806 to 0.949)	0.01±0.01
ISV	2.81±5.95	2.90±5.36	2.95±6.71	3.05±5.88	0.943 (0.894 to 0.974)	2.96±2.56
IVA	0.03±0.08	0.03±0.07	0.02±0.10	0.02±0.09	0.881 (0.787 to 0.944)	0.06±0.05
IHA	-1.56±15.04	-3.67±14.09	-2.20±16.52	-4.30±12.92	0.573 (0.363 to 0.767)	20.53±16.66
IHD	0.00±0.01	0.00±0.01	0.00±0.01	0.00±0.01	0.816 (0.683 to 0.910)	0.01±0.01
Kmax = maximum keratometry; D = diopter; Kmin = minimum keratometry; CCT = central corneal thickness; MCT = minimum corneal thickness; ACD = anterior chamber depth; KI = keratoconus index, CKI = center keratoconus index; ISV = index of surface variance; IVA = index of vertical asymmetry; IHA = index of height asymmetry; IHD = index of height decentration						

## Discussion

Accurate measurements of keratometry and corneal thickness is essential in keratoconus management and the evaluation of CXL effectiveness [**13**,**14**]. To date, several studies have been done on the reliability of various Pentacam indices in keratoconus patients [**8**,**10**,**15**] and even after CXL [**16**,**17**]. However, the effect of CXL on the repeatability of Pentacam measurements has not been studied yet. The purpose of our study was to determine whether corneal changes after CXL affect the repeatability of Pentacam measurements of keratometry, corneal thickness, and keratoconus indexes. Baseline and post-CXL ICC values for all indices were above 0.9, but much lower for the measures of change. Similarly, the RI values of the measures of changes were lower compared to intra-session RI of each index.

ICC or test-retest reliability reflects the variation of measurements made under similar conditions over a short period of time. Lack of device calibration, low device quality, erroneous measurement methods, and technician inexperience can lead to reduced ICC. For this reason, in our study, the same technician performed all baseline and post-CXL measurements to eliminate the effect of operator’s experience and keep the focus on the precision of the device and the effect of CXL on this precision. An ICC above 0.9 was indicative of excellent device reliability [**18**] for the anterior and posterior corneal indices. In other words, the repeatability of posterior keratometry after CXL was comparative to anterior keratometry and similarly reliable. Labiris et al. [**17**] also reported ICC values above 0.9 for posterior elevation indices in KCN and post-CXL groups. In the study by Sideroudi et al. [**9**], the repeatability of K1, K2, and Q-value of the anterior cornea in keratoconus and post-CXL cases was greater than 0.9. A strength of our study was that we compared the reliability of KCN and post-CXL longitudinally in the same group before and after CXL. Results indicated that CXL does not significantly affect the repeatability of Pentacam measurements. The lower ICC seen with the four measures of change can be due to variations in the response to CXL. In this regard, the repeatability values of the measures of change in posterior indices were much lower than anterior indices.

Although the limited sample size of this study reduced the power of comparative tests, the design of the study (i.e. before-after) allowed an intra-individual comparison of the repeatability of the indices. Therefore, despite changes in the cornea after CXL, the repeatability values of the measured indices with Pentacam were not affected by this procedure, and they did not differ from baseline values. However, the high variance of the IRs calculated for the measures of changes indicated that inter-study differences should be interpreted with caution and the repeatability of measures of change should be taken into consideration. This is especially important for systematic reviews because inter-study differences are partly due to differences in treatment response and partly due to low repeatability of measures of change.

**Funding**

None.

**Conflict of interest**

All authors certify that they have no affiliations with or involvement in any organization or entity with any financial interest or non-financial interest in the subject matter or materials discussed in this manuscript.
